# Dr. Mackie's Account of a Case of Ossified Uterus

**Published:** 1800-05

**Authors:** Arthur Mackie

**Affiliations:** Lewisham


### *To the Editors of the Medical and Phyfical Journal.*

Gentlemen,

I Tranfmit to you a fingular cafe of Offified Uterus, which I truft you may think worthy of a place in your very interefting Journal; and am, with much refpect,

Gentlemen,

Your moft obedient fervant,

*Lewifham,*

*April* 3, 1800.

ARTHUR MACKIE, M. D.

---

ON the 28th of January laft I was called on to open the body of Mrs. Ruff, who died the day before, in the 72d year of her age.

She had fuffered much from difeafe, and defired that fhe might be opened after her death. What I could learn of her hiftory was as follows:—She was born of healthy parents, and was herfelf ftrong and healthy till her marriage in her 32d year. She foon after was fuppofed to become pregnant; but fymptoms of dropfy alfo appearing, fhe was attended by Drs. Fothergill and Watfon, and an accoucheur, who all agreed that it was a cafe of pregnancy, (though her menfes continued regularly) accompanied by anafarca; and they prefcribed fome diuretic medicines. Thefe produced a copious difcharge of urine; the dropfical dropsical appearances subsided, but the size of the abdomen continued to increase, with more than usual uneasiness, till the supposed time of her delivery.

This anxious period at length passed over, and to the great alarm of the patient and her family, no labour or delivery took place. From this time she had bad and irregular health; her bulk continued undiminished; she had obstinate constipations; she was frequently attacked with hot, burning, excruciating pains in her belly, that could only be relieved by opium; she became subject to violent gouty paroxysms, which confined her to her bed two or three months in the year, and left her extremities much distorted; and for a long while past, she had been a constant sufferer from the size and increased weight of the abdomen; and, particularly, on turning in bed, when the bulk fell to the lower side, in a lump, (as she expressed it) it never failed to give her the most distressing pain and uneasiness. A paralytic stroke, which deprived her of the use of speech, and of one side, preceded her death a few days.

On uncovering the abdomen, it appeared unusually large, hard, and incompressible; the bulk rather exceeding that of a pregnant woman at her full time, and inclining to the left side. In cutting along the linea alba, after dividing the skin, I found the muscles so much ossified, from about three inches above the umbilicus, that I could not separate them without a very strong knife. The ossification extended as much below the navel, and about three or four inches on each side, having some resemblance to the top of a child's skull. The inside had a smooth, shining polish, apparently from its friction on the uterus, between which and these ossified muscles nothing intervened; the peritonæum being entirely obliterated. On laying aside the separated integuments, the uterus appeared in full view, filling almost the whole cavity of the abdomen, and even pressing against the diaphragm. Its appearance somewhat resembled a very large pellucid bladder distended with hog's lard, and pressed a little flat at the fundus. It was some time before I observed the intestines, which were pushed into a space not exceeding a cube of two inches, in the lower part of the left side of the pelvis, and seemed crowded, shrunk, and empty. The omentum was obliterated. Near them lay above twenty hydatids of different sizes, of a beautiful polished white colour, and several of them as large as pullets eggs, but without any fluid.

On attempting to cut into the uterus, the knife made no impression, till great force was used, the ossification was so complete: the divided sides appeared of a chalky, brittle, dead white; no vestige of nerves or blood vessels remaining. The fundus was about a quarter of an inch thick, and perfect bone; the body of the uterus thicker, and not unlike the thickeft parts of the occiput. The offification ended at the cervix, which was of a hardifh febaceous texture, and four or five inches thick. On cutting through this part, about a quart of ftrongly fœtid pus was difcharged; and on making a complete divifion of the uterus, (which was large enough to contain the body of a full grown fœtus) I found a large detached unformed fpungy mafs, compofed of foft and hard parts; the former refembling moift decayed wood, and eafily compreffible into a fmall bulk; the latter fomewhat like the ends of the long bones, but no fpecific part afcertainable; though the whole conveyed to my mind the poffibility of its once having been a fœtus.

The uterus with this fubftance, when taken out of the body, weighed eighteen pounds three-quarters, avoirdupois.

The ovaria, Fallopian tubes, and ligamenta rotunda, differed little from their natural fize and colour; the liver was fmall, hard, and of a dark red, and pufhed backwards; the gall-bladder appeared as if fqueezed by the uterus, part of which was ftained with bile. The kidneys were larger than ufual, and covered with watery blifters; the vefica urinaria fmall and empty; the ftomach, fpleen and pancreas appeared fhrunk, and with the diaphragm pufhed upwards.

I was not allowed time to make a more minute examination, or any further remarks; I thought thefe fufficiently curious to merit being recorded.

